# Human Age Estimation Method Robust to Camera Sensor and/or Face Movement

**DOI:** 10.3390/s150921898

**Published:** 2015-08-31

**Authors:** Dat Tien Nguyen, So Ra Cho, Tuyen Danh Pham, Kang Ryoung Park

**Affiliations:** Division of Electronics and Electrical Engineering, Dongguk University, 30, Pildong-ro 1-gil, Jung-gu, Seoul 100-715, Korea; E-Mails: soracho@dongguk.edu (S.R.C.); phamdanhtuyen@gmail.com (T.D.P); parkgr@dongguk.edu (K.R.P.)

**Keywords:** affective interface for entertainment, human age estimation, blurring effect of camera sensor

## Abstract

Human age can be employed in many useful real-life applications, such as customer service systems, automatic vending machines, entertainment, *etc.* In order to obtain age information, image-based age estimation systems have been developed using information from the human face. However, limitations exist for current age estimation systems because of the various factors of camera motion and optical blurring, facial expressions, gender, *etc.* Motion blurring can usually be presented on face images by the movement of the camera sensor and/or the movement of the face during image acquisition. Therefore, the facial feature in captured images can be transformed according to the amount of motion, which causes performance degradation of age estimation systems. In this paper, the problem caused by motion blurring is addressed and its solution is proposed in order to make age estimation systems robust to the effects of motion blurring. Experiment results show that our method is more efficient for enhancing age estimation performance compared with systems that do not employ our method.

## 1. Introduction

Human age estimation has many useful applications, such as face recognition systems that are robust to age progress, evaluation systems of the effectiveness of advertising to customers, and systems that help prevent minors from buying alcohol, tobacco, or accessing adult websites [1,2]. Because of its useful applications, age estimation has become an attractive research area, and it has been studied intensely. In most previous studies, human age has been estimated using facial images. This type of method uses differences in the appearance of facial regions between old and young people. Several methods have been proposed for this problem [3,4,5,6,7,8,9], and the popular method is based on active appearance models (AAMs) [3,4,5]. This method models the shape of the human face using multiple landmark points that describe the shape of the face. In addition, the appearance of the face is also modeled using principal component analysis (PCA). However, many landmark points should be detected in order to describe the shape of the face, and detection performance can be affected by head movement, complex backgrounds, and head pose. In addition, the detection of multiple landmark points requires significant processing time, thus making it difficult to apply to real-time systems. Therefore, methods that do not use AAM are proposed [7,8,9] that do not require exact detection of the landmark points on a facial area. These methods extract age features from the facial region without accurate positions for the landmark points.

In addition, methods have been proposed for extracting high-frequency components from the face region and/or extract the appearance of special skin textures that appear on the face region when people age [6,7,8,9]. The facial features used for age estimation are classified into three categories: local, global, and hybrid. Typical local features are wrinkles, skin, and hair. In previous research, facial images are classified into three age groups: babies, young adults, and senior adults based on the features of the distance ratio of facial components and the wrinkle features [10]. Txia *et al.* proposed an age classification method based on hair color and wrinkle features obtained by the Sobel edge operator [11]. Global features show the overall characteristics of the face area for age estimation, which are based on AAM, Gabor wavelet transform (GWT) [12], and subspace features based on image intensity [13]. As the third method, hybrid features based on a combination of global and local features are used in previous research [14]. These extracted features are then inputted to regression or classification machines in order to estimate human age [1,2,3].

Classification steps are performed for age estimation with the age features. This can be classified into three approaches: age group classification [12], single-level age estimation [5], and hierarchical age estimation [3,5,15]. The first approach is the method that approximately predicts an age group, instead of estimating accurate age. The second and third approaches focus on estimating the accurate age. Among these two approaches, single-level age estimation is used to estimate an accurate age in the entire data set without pre-classification. In this case, age estimation accuracy can be reduced because there are many data sets to classify. To overcome this problem, hierarchical age estimation is proposed, which is a coarse-to-fine method, with pre-classification, and this produces improved performance [3,5,15]. This is because age estimation on a smaller group can simplify the complexities of classification and computational load [15].

Although these previous methods can produce good estimation results, they still have many challenges. Some such challenges include the effect of gender differences, facial expressions, or the quality of captured images on age estimation. For example, at the same age, the female face normally appears younger than the male face [7]. Another factor is facial expressions. By presenting feelings, the appearance of the human face changes in both texture and shape.

In addition, in most of the previous methods, the authors used only focused and good quality images for age estimation. Consequently, when poor quality images are used in the system, the estimation results become untrustworthy. Blurring is one of the major factors that cause poor quality in face images. By including blurring effects on face images, both the shape and texture information of the face are changed and/or lost. There are two types of blurring, optical blurring of the camera and motion blurring caused by the relative movement of the camera and observed objects. Although optical blurring can be compensated through an algorithm for auto focusing, motion blurring is frequently present on an image because of the natural and random behavior of humans or camera movements. As a result, the captured image becomes blurred, and this causes degradation of the age estimation performance. However, to the best of our knowledge, there is no previous research that considers the effects of motion blurring on age estimation systems.

In other researches, they used deep convolutional neural network (CNN) [16] and CNN with support vector machine (SVM) [17] to extract the features and train the model and to classify the input image to specific group of age and gender. Their methods could be used for unfiltered image, internet images and its performance is superior to previous research. However, their methods require complex architecture and time consuming procedure for training the network. In addition, this research is just for age classification not precise age estimation.

In [18], they proposed the age estimation using feature extraction method based on multi-scale CNN. In previous research [19], they proposed the method of estimating the human age using feature extraction method based on CNN and age classification/estimation based on SVM/support vector regression (SVR). Although the performance of their age estimation is superior to previous methods, they require complex architecture and time consuming procedure for training the network [18,19]. In addition, landmark detection based on active shape model (ASM) is affected by background and non-uniform illumination, and it takes much processing time [18].

To overcome the problem of previous age estimation systems on poor quality images caused by motion blurring effects, we propose an age estimation method that is robust to the effects of motion blur. Our research is novel in the following four ways compared to previous methods. First, we propose a method for estimating motion blur parameters (the direction and amount of motion blur) based on the modified Radon transform with *ρ* range-based summation and fitting method. Second, the input facial image is pre-classified into one of the several groups of motion blur based on the estimation results of the motion blur parameters, which can reduce the variation of facial images caused by motion blurring in each group. Third, an up-to-date age estimation system based on multi-level local binary pattern (MLBP), Gabor filtering, PCA, and SVR is used to estimate human age. Fourth, an appropriate age estimator is applied for each group of motion-blurred images. Using this scheme, the age estimator used for each group of motion-blurred images can efficiently describe the age characteristics of the images in that group. Consequently, age estimation performance can be enhanced greatly, even with an image that includes motion blurring.

Table 1 lists the comparisons of previous and proposed studies on age estimation.

**Table 1:** Summary of previous and proposed studies on age estimation.

Category	Method	Strength	Weakness
Age estimation not considering motion blur effect	-AAM [3,4,5] or non-AAM [7,8,9] based methods, local features [10,11], global features [12,13], hybrid feature [14]-based methods, age group classification [10,12], single-level age estimation [5], hierarchical age estimation [3,5,15], deep CNN-based methods [16,17,18,19]	-Produce good estimation results with clear and good quality input images	-Estimation accuracy is degraded significantly with motion blurred images
Age estimation considering motion blur effect (Proposed method)	-Motion blur parameters are estimated based on the modified Radon transform with *ρ* range-based summation and fitting method -Input facial image is pre-classified into one of several groups of motion blur based on estimated motion blur parameters -An appropriate age estimator is applied for each group of motion-blurred images	-Robust to image motion blurring	-Additional procedure for estimating motion blur parameters for image is required

The remainder of this paper is structured as follows: in Section 2, we describe the proposed age estimation method. Then, the experimental environment and results are shown in Section 3. Finally, we present the conclusions in Section 4.

## 2. Proposed Method for Human Age Estimation Robust to Motion Blurring Effects

### 2.1. Proposed Method Overview

**Figure 1:**
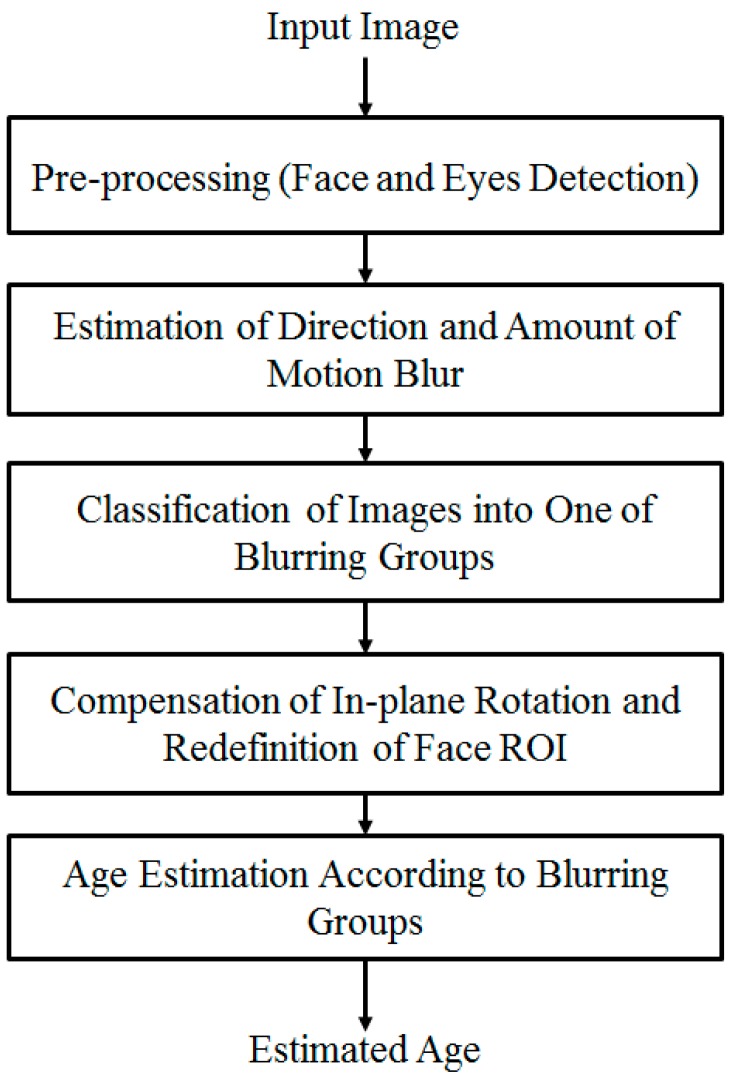
Overall procedure for our method.

The overall procedure for our method of the human age estimation system that is robust to the effects of motion blurring is depicted in Figure 1. As shown in the figure, we first perform a preprocessing step in order to localize the face and eye region in the input face image. This step is necessary for face region localization and removal of the background regions in the input images. The details of this step are explained in Section 2.2. With the detected position of the face and eye regions, we approximately define the face region of interest (ROI) to classify the focused and motion blurred images using a method based on the modified Radon transform with *ρ* range-based summation. In the case of images that contain motion blurring, we further estimate the parameters of motion blurring, including motion direction and amount of motion blur, using the modified Radon transform and fitting methods. In this research, we assume that motion blur is in the form of linear motion blurring. In Section 2.3, we provide more details of our method for this step.

From this step, we obtain the estimated parameters of motion direction and the amount of motion blur in a motion-blurred image. Using these parameters, we pre-classify the input facial images into one of several groups of motion blurring, such as the focused group, which contains only the focused images and trivially blurred images, and the blurring groups that contain images at higher degrees of motion parameters (motion direction and motion length). As a result, we obtain several groups of facial images where image variation caused by motion blurring is in a small range.

Finally, in order to enhance the performance of the age estimation system and obtain the appropriate age estimator, we perform a training procedure with each group of motion-blurred images using an age estimation method based on MLBP, Gabor filtering, PCA, and SVR. Because each group of motion-blurred images contains the images from a small range of motion blur, face variation is small. Consequently, the age estimator trained with the images of that group can well describe the characteristics of the images in that group. The explanation of the age estimation method is presented in Section 2.4.

### 2.2. Pre-Processing Steps for Human Face Detection and in-Plane Rotation Compensation

**Figure 2:**
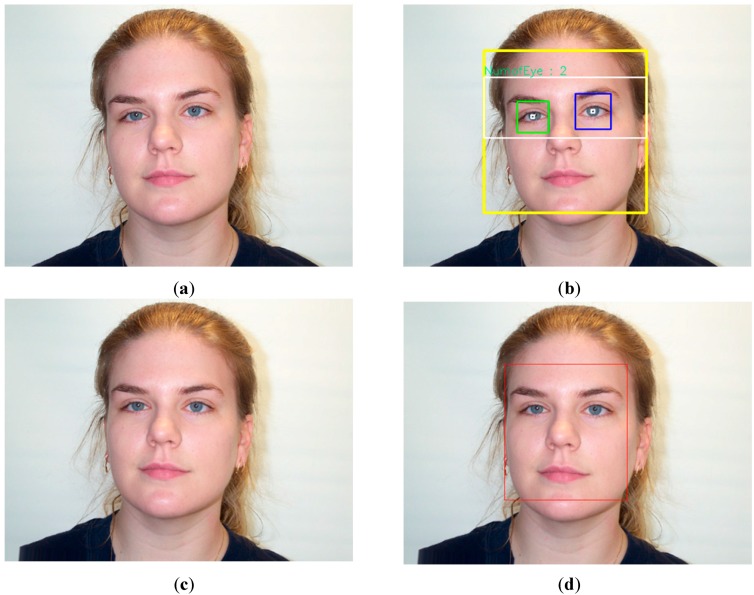
Demonstration of in-plane rotation compensation and face region redefinition in our method: (**a**) input facial image; (**b**) detection results of face and eyes using Adaboost method; (**c**) in-plane rotation compensation; and (**d**) redefinition of face region.

In normal cases, the captured face images can contain both a human face and background regions, as shown in Figure 2a. Because there is no age information in the background region, it should first be removed before executing further processing steps. For this purpose, we perform a pre-processing step to detect the location of the face and the position of the two eyes using the adaptive boosting (Adaboost) method [20]. In order to detect the face from the facial image, the Adaboost method extracts the face feature from the input facial image and constructs several weak face classifiers. Finally, a strong face classifier is built by combining these weak classifiers using adaptive boosting method. The same method is applied to detect the eyes region by applying the Adaboost eye classifier on the detected face region. In Figure 2b, we show an example of face and eyes detection by the Adaboost method.

Age estimation performance can be affected by misalignment of the facial region [21]. Therefore, our method compensates the in-plane rotation of the facial region using the detected position of the two eyes, as shown in Figure 2b. In general, in-plane rotation can occur because of human head pose during image acquisition. Based on previous research [7], we compensate the in-plane rotation through rotating the facial region by angle θ calculated by Equation (1). In Equation (1), (*R_x_*, *R_y_*) and (*L_x_*, *L_y_*) represent the detected positions of the right and left eyes, respectively. By rotating the face region by angle θ, the face regions are aligned. As a result, the face region can be estimated efficiently; then we attempt to capture as much face information as possible, and remove as much background and noise regions as possible. In Figure 2c, we show a sample result of in-plane rotation compensation. Based on the position of the two eyes and the result of in-plane rotation compensation, we redefine the face region to make it fit the face region, as shown in Figure 2d. Because the face region is redefined to fit with the correct face region, instead of face ROI detected by the Adaboost method, the redefined face ROI contains richer age information than the face ROI detected by the Adaboost method. Consequently, age estimation performance can be enhanced.

(1) $$\theta =\;tan^{-1}\left(\frac{R_{Y}-L_{y}}{R_{x}-L_{x}}\right)$$

### 2.3. Proposed Method for Estimating Motion Blur Parameters

#### *2.3.1. Motion Blur Modeling and Its Point-Spread Function* 

The quality of captured images can be affected by many factors, such as the capturing conditions, environment, and capturing devices. Motion blur is a common type of image quality degradation caused by the relative motion between the camera and observed objects [22,23,24,25,26,27]. Similar to the optical blurring of camera sensors, motion blurring makes captured images to appear blurred, and changes the image’s texture according to the motion direction and amount of motion (motion length). Consequently, it causes degradation in the image quality and performance of image processing systems. In order to manage motion blurring, it is usually modeled by the term of the point-spread function (PSF) [22]. By presenting the PSF term, the image observed under motion blurring is modeled by Equation (2). In this equation, the observed image *g*(*x,y*) is obtained by convolution of the original scene *f*(*x,y*) and the motion blur PSF function *h*(*x,y*), where *x* and *y* are the horizontal and vertical positions of image pixel, respectively. In addition, the noise term η(x,y) is added to produce the image observed in the actual case. The symbol “*” indicates the convolution operation in this equation. In the frequency domain, Equation (2) is represented by Equation (3):

(2) g(x,y)=f(x,y)*h(x,y)+η(x,y)

(3) G(u,v)=F(u,v)×H(u,v)+N(u,v)

In general, it is extremely difficult to manage motion blurring because of the types of motion blurring and the effects of noise. In our research, we consider the common general type of motion blurring, called linear motion blurring, and the PSF is given by Equation (4) [22]:

(4) $$h\left(x,y\right)=\{\begin{array}{c} \frac{1}{L}\;if\;\sqrt{x^{2}+y^{2}}\leq \frac{L}{2}\;and\;\frac{x}{y}=-tan\left(\theta \right)\\ 0\;otherwise \end{array}$$

where, *L* is the amount of motion blur, called motion length, and θ is the motion direction. In the frequency domain, the PSF function of motion blur is given by Equation (5) [22]. In addition, if we neglect the noise term, the consequent observed image in the frequency domain in Equation (3) is reduced to Equation (6). For calculation purposes, the power-spectrum of images in the frequency domain is given by Equation (7) using the log operator:

(5) $$H\left(u,v\right)=\frac{\text{sin}\left(\pi L\left(ucos\theta +vsin\theta \right)\right)}{\pi L\left(ucos\theta +vsin\theta \right)}$$

(6) G(u,v)=F(u,v)×H(u,v)

(7) log(G(u,v))=log(F(u,v))+log(H(u,v))

**Figure 3:**
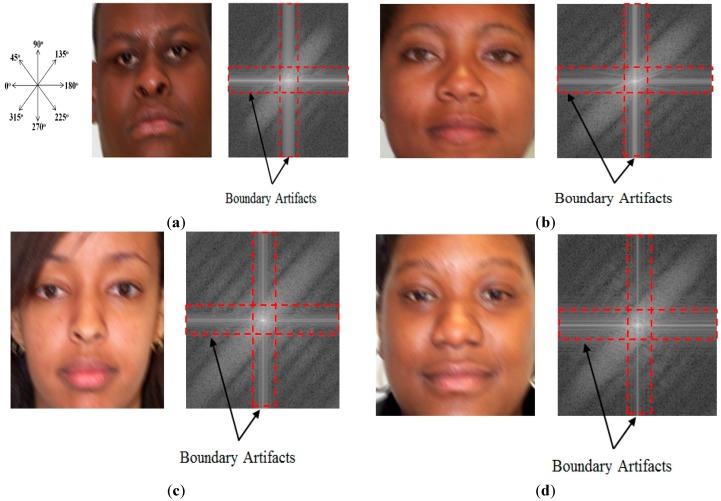
Examples of motion blurred images and their corresponding power spectrums with motion length of 7 and motion direction of 45°: (**a**) the first case of motion blurred image and its corresponding power spectrums with the indicator of motion direction; (**b**–**d**) the other cases of motion blurred images and their corresponding power spectrums.

In Figure 3, we show some examples of motion-blurred images and their corresponding representations in the frequency domain. It can be observed from Equation (6) and Figure 3 that the power spectrum of motion-blurred images has a directional characteristic by presenting dominant parallel lines that are orthogonal to the motion blur direction. This is because the edge elongates in the direction of motion blur in the image, which increases the high frequency components in the orthogonal direction to the elongated edge direction. This characteristic plays a key role in estimating the parameters of motion blur.

**Figure 4:**
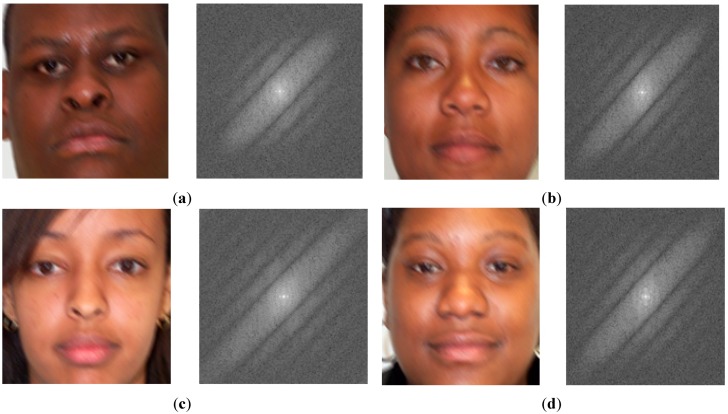
Implementation result examples for Hann windowing method for removing boundary artifacts on images in Figure 3: (**a**–**d**) the four examples of results by Hann windowing method.

As shown in Figure 3, the power spectrum of motion-blurred images contains a boundary artifact in the horizontal and vertical frequency axes caused by the image boundaries. It is easy to observe that the boundary artifact reduces the directional characteristic of the power spectrum of the motion-blurred image. Therefore, the estimation performance of the motion blur direction and motion length is reduced. In order to remove the boundary artifact, we use the Hann windowing method [22]. The 1-D Hann window is defined in Equation (8). In this equation, *N* is the size of the Hann window. By applying the Hann windowing method in both the horizontal and vertical directions, we make the image to become a periodic signal and remove the boundary artifact of the image. Examples of the results by Hann windowing are given in Figure 4. As shown in Figure 4, using the Hann windowing method allows us to efficiently remove the boundary artifacts on the images and make the directional characteristics become clear:

(8) $$W\left(x\right)=\frac{1}{2}\left[1-\text{cos}\left(\frac{2\pi x}{N}\right)\right]$$

#### *2.3.2. Motion Direction Estimation Based on Modified Radon Transform with ρ Range-Based Summation* 

**Figure 5:**
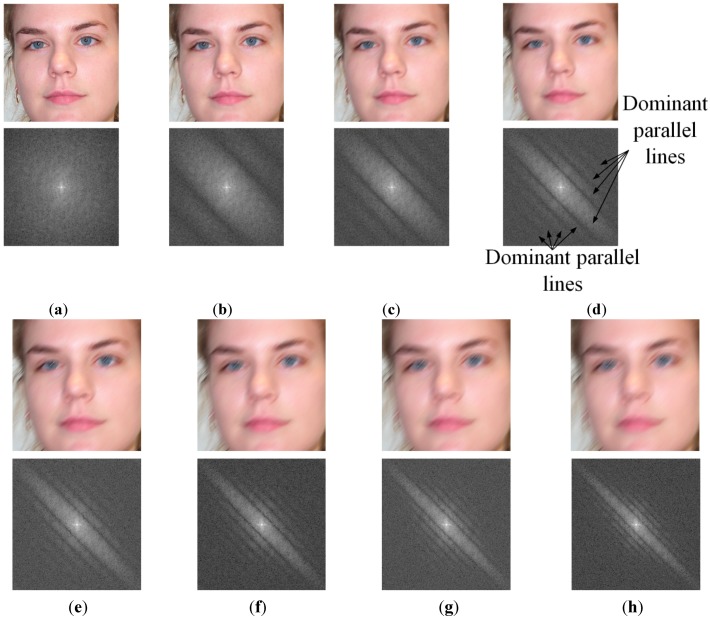
Examples of motion-blurred images (upper Figures of **a**–**h**) and corresponding power spectrum (lower Figures of **a**–**h**) with various amounts of motion blur and motion directions: (**a**) case without motion blurring; (**b**–**h**) cases of motion blurring with motion length from 3 to 15 with step of 2, respectively, and motion direction of 135°.

In order to estimate the motion blur parameters, motion direction must be estimated first. In previous studies, motion direction is estimated using the directional characteristic as shown in Figure 4. Several methods have been used, such as Hough transform, Radon transform, and Steerable filters [22]. The idea of Hough transform-based methods is that they attempt to detect lines in the power spectrum of blurred images, and choose the direction that is orthogonal to the longest line as the direction of motion blurring. Intuitively, this method works well because of the directional characteristic of the power spectrum of motion-blurred images. However, this method has several limitations, the largest of which is the calculation of the edge map (binarization map) of the power-spectrum image. According to the amount of motion blur and motion direction, the size of the dominant parallel lines is different, as shown in Figure 5. In addition, from Equation (6), it could be desired for the length of the dominant parallel lines to also be dependent on the number and distribution of the high frequency components in the focused image (scene image without blurring effect and noise). Consequently, the threshold for the binarization step can be varied according to the motion direction and amount of motion blur. Therefore, performance of the method depends greatly on the threshold for binarization of the power-spectrum image.

**Figure 6:**
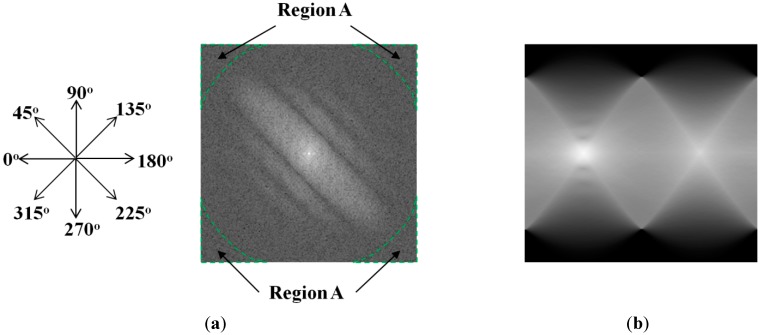
Example of Radon transform method: (**a**) input power-spectrum image; (**b**) Radon image in all directions (horizontal and vertical axes are θ and ρ of Equation (9)).

Another method for direction estimation is the Radon transform-based method [22,23]. This method applies the Radon transform on the power-spectrum image to find the dominant parallel lines by finding the maximum peak of the Radon transform image. Because the power-spectrum image is used instead of the binarized image, the Radon transform-based method overcomes the limitations of the Hough transform-based method on the binarization step. However, this method has also its own limitations. Because this method calculates the integral values of the image pixels along the projection directions, it has the problem of the difference of the frequency areas where the pixels are taken in each direction. For example, in Figure 6a, the pixels are taken from the additional frequency area (region A) in the case of the diagonal direction (45°, 135°, 225° and 315°) compared to the horizontal (0° and 180°) and vertical (90° and 270°) directions. Therefore, finding the maximum peak cannot ensure good estimation results for motion direction. In addition, when the amount of motion blur is small, the directional characteristic is not clear, which can also cause wrong estimation for the motion direction.

In order to overcome the limitations of previous methods on motion direction estimation, we propose a new method for direction estimation of motion-blurred images. In this method, we modify the Radon transform to overcome the limitation of the traditional Radon transform method by taking the statistical characteristic of dominant parallel lines.

The Radon transform is an efficient image transformation method widely used in medical image processing systems. This method produces 1-D image data from normal 2-D image data by projecting the 2-D image data along a specific direction. Figure 6 shows a visualization of the Radon transform applied on a power-spectrum image. Using a mathematical expression, the Radon transform is expressed by Equation (9). In this equation, δ(x) indicates the delta function, f(x,y) is the input image (Figure 6a), θ is the projection direction, and ρ is the distance:

(9) $$R\left(\rho ,\theta \right)=\overset{M-1}{\underset{x=0}{\sum }}\overset{N-1}{\underset{y=0}{\sum }}f\left(x,y\right)\delta \left(xcos\theta +ysin\theta -\rho \right)$$

In Figure 6, it can be observed that because of the problem of the number of pixels taken in the projection directions, the Radon image contains several similar peaks, and finding the maximum peak is not efficient for accurate direction estimation. In order to overcome the limitation of the traditional Radon transform method, we propose the modified Radon transform method using only the image region inside a circle of the power-spectrum images, instead of using the entire image.

**Figure 7:**
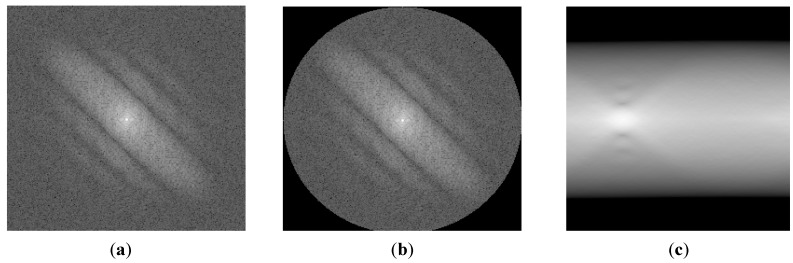
Demonstration of selected area for Radon transform in our research: (**a**) original power spectrum of a motion-blurred image; (**b**) selected region for motion direction estimation, and (**c**) Radon transform of selected region (horizontal and vertical axes are θ and ρ of Equation (9)).

As explained in Equation (6), the power-spectrum image of motion blur is formed by multiplying the power spectrum of the focused image (the scene image without blur effect and noise) with a sinc function. The center position of the power-spectrum image indicates the low frequency components, whereas the positions far from the center indicate the high-frequency components. Normally, the very high-frequency components in an image are smaller than the lower frequency components. In addition, by multiplying the power spectrum with the sinc function, the high-frequency components become small values. Consequently, the directional characteristic is mainly concentrated on the region around the low frequency components, as shown in Figure 7a. Based on this observation, in order to solve the problem of the traditional Radon transform method, we only perform the Radon transform on the image area inside a circle, as shown in Figure 7b. The resulting Radon image for Figure 7b is given in Figure 7c.

As explained before, because of the distribution of the high frequency components of the focused image and the amount of motion blur, the dominant parallel lines of the power spectrum of motion blur could not be in the correct motion blur direction. In order to obtain a more correct direction for motion blur, we propose the method of ρ range-based summation, as shown in Equation (10). In this equation, “margin” is the margin value of  ρ. The image obtained by this method corresponds to the summation of the modified Radon transform image at each motion direction with a filter size of (2 × margin + 1). Consequently, we can estimate the noise effects, random distribution of the high frequency components of the focused images, and effects of the amount of motion blur. In another interpretation, the modified Radon transform with ρ range-based summation takes the statistical characteristic of the dominant parallel lines in order to produce a more accurate estimation result by accumulating the power spectrum values inside a larger region, instead of a single region, as with the traditional Radon transform-based method. Finally, the direction of motion blur is estimated by finding the largest value (peak value), as shown in *C* of Equation (11), and taking the orthogonal value. The overall procedure for estimating the motion blur direction in our research is given in Algorithm 1.

(10) $$P\left(\theta \right)=\overset{margin}{\underset{\rho =-margin}{\sum }}R\left(\rho ,\theta \right)$$

(11) $$C=\underset{\theta^{'}}{\arg\max(P(\theta \text{))}}$$

**Algorithm 1**: Motion Direction Estimation Using Modified Radon Transform with *ρ* range-based SummationConvert blurred image into gray-level imagePerforming Hann windowing to remove the boundary artifactsTransform the image in step 2 from spatial domain to frequency domain using Fourier transform to obtain the image in frequency domain *F*(*u,v*)Compute the log of the power spectrum of *F*(*u,v*) in step 3 and remove the very high frequency components, as shown in Figure 7bCompute *ρ* range-based summation using Equation (10)Find the largest value (peak value), as shown in *C* of Equation (11), and indicate the direction of the dominant parallel line


#### 2.3.3. Estimation of Motion Blur Amount (Motion Length)

**Figure 8:**
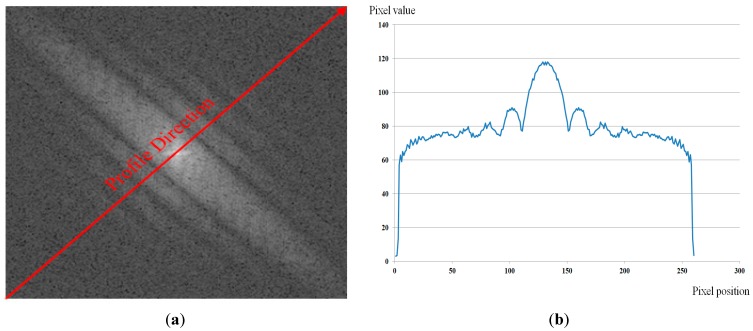
Example of profile in motion direction where motion direction is 135° and amount of motion blur is 9: (**a**) power-spectrum image; and (**b**) profile in motion direction.

As shown in Equation (6), by neglecting the noise term, the representation of motion-blurred images in the frequency domain is the multiplication of the focused image in the frequency domain and a sinc function. By taking the log of Equation (6), the power spectrum of the motion-blurred image is the summation of the log of the power spectrum of the focused image and the motion blur kernel. Because the power spectrum of the motion blur kernel is in the shape of the sinc function, the profile of the power spectrum in the motion direction (that is orthogonal to the dominant direction of the power-spectrum image) also has the sinc function shape [22,23]. In Figure 8, we show an example of the power spectrum profile in the motion direction.

In order to estimate the amount of motion blur, two general methods have been used in previous studies, including the Radon transform-based and Cepstral-based methods. The Radon transform-based method extracts the profile in the motion direction of the power-spectrum image and attempts to find all local minimum points of the profile. Then, the average distance between these local minimum points is measured and used to calculate the amount of motion blur [22]. Another way to estimate the amount of motion blur is to use the Cepstral transform based on the inverse Fourier transform of the log of the power spectrum of motion-blurred images. However, these methods have the effects of noise and frequency distribution in focused images. Because of these factors, the extracted profile is not identical in the sinc shape; it is simply a sinc-like shape, as shown in Figure 8b. Consequently, estimation errors can occur.

**Figure 9:**
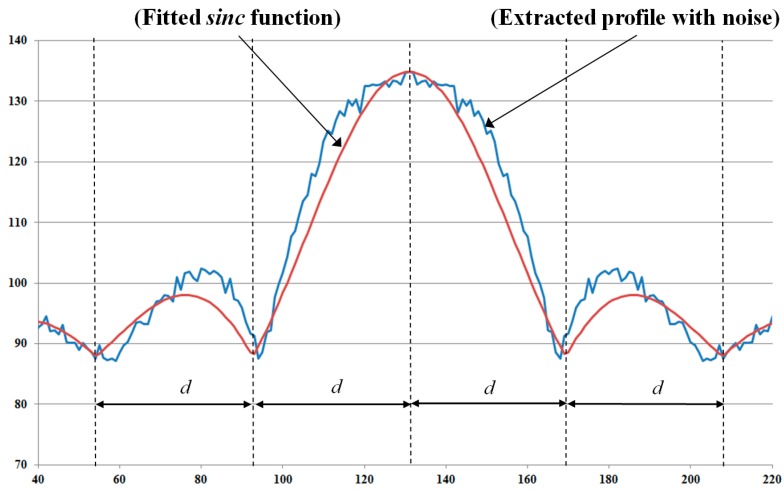
Example of fitting process to find best-fitted sinc function to an extracted profile.

To overcome the limitations of previous methods, we propose a method for estimating the amount of motion blur using a fitting method. Because of the effects of noise and the distribution of frequency components of the focused image, the extracted profile in the motion direction of power-spectrum images is a sinc-like shape, instead of a sinc shape, as shown in Figure 8b. Therefore, instead of finding the local minimum points in the profile or making the inverse Fourier transform, we perform the fitting method to find the best-fit sinc shape to the extracted profile. The fitting process is performed by choosing the sinc function that minimizes the error between the extracted profile and selected sinc function. By estimating the best-fitted sinc function to the extracted profile, we estimate the distance (Figure 9d) based on the local minimum points from the fitted function. Finally, the amount of motion blur in the image is estimated as N/d where N is the horizontal or vertical length of image [22]. In Figure 9, we show an example of the fitting process to find the best-fitted sinc function to the extracted profile. In addition, the overall algorithm for estimating the amount (length) of motion blur in our research is given in Algorithm 2.

**Algorithm 2**: Estimation of the Amount (Length) of Motion BlurEstimate the motion blur direction using Algorithm 1.Extract the intersection profile in the motion blur direction of the power-spectrum imagePerform the sinc fitting process to approximate the parameter of the sinc function and calculate the value of distance *d*.For an image of size *N × N*, the motion length is calculated by *N/d*


#### 2.3.4. Proposed Focus Score Measurement for Classification of Focused and Motion-Blurred Images

**Figure 10:**
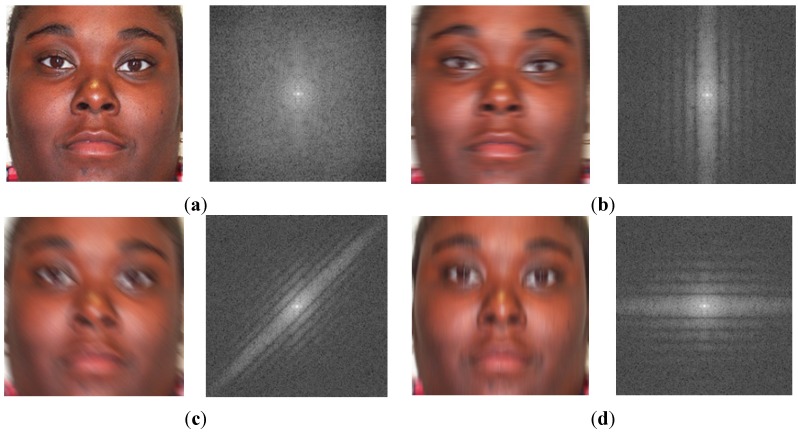
Example of power-spectrum images of focused and motion-blurred images at different motion blur directions: (**a**) focused image without motion blurring; (**b**) motion blur with direction of 0° and motion length of 15; (**c**) motion blur with direction of 45° and motion length of 15; (**d**) motion blur with direction of 90° and motion length of 15.

In addition to the method for estimating motion blur parameters, we also propose a focus score measurement for classifying the focused and motion-blurred images. In Figure 10, we show an example of the power-spectrum images of a focused image and the power spectrums of motion-blurred images with different direction values and amount of motion blur (motion length).

As shown in Equation (6) and Figure 10, because the focused image has no motion blur effect, its power spectrum does not contain the dominant parallel lines, as does the power spectrum of motion-blurred images. In the case of motion-blurred images, Figure 10 shows that the gray-level of the pixels along the motion blur direction in the power-spectrum images is much lower than that of the orthogonal direction. Consequently, the difference between the total gray-level of the image pixels in the dominant and non-dominant directions becomes very large.

In addition, because of non-directional characteristics, the power spectrum of the focused image only has the characteristic where the difference between the total gray-level of the image pixels in the dominant and non-dominant directions becomes very small. Based on this characteristic, we propose a focus score measurement for classifying the focused and motion-blurred images as shown in Equation (12). In this equation, $S_{\theta }$ indicates the sum of the gray-levels of the power spectrum along the dominant direction, and $S_{\theta +90}$ indicates the sum of the gray-levels of the power spectrum along the orthogonal direction of θ. Using the proposed focus score measurement in Equation (12), the focus scores of the focused images tend to be close to 100, whereas the focus scores of the motion-blurred images become lower because of the directional characteristic:

(12) $$FS=100\times \frac{S_{\theta +90}}{S_{\theta }}$$

**Figure 11:**
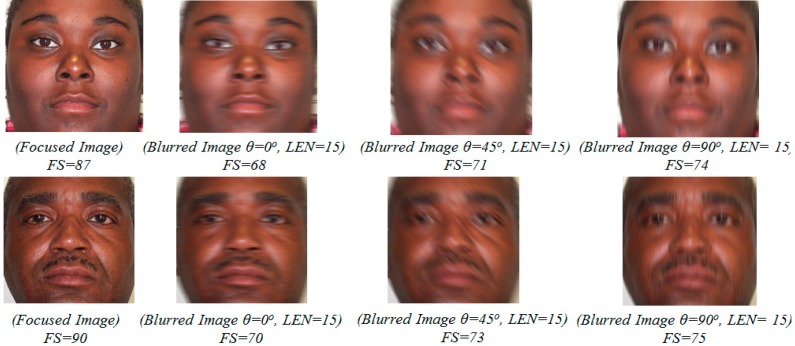
Example of focus score measurements using our method with focused image and corresponding motion-blurred images (θ is motion direction; *LEN* is amount (length) of motion blur).

**Figure 12:**
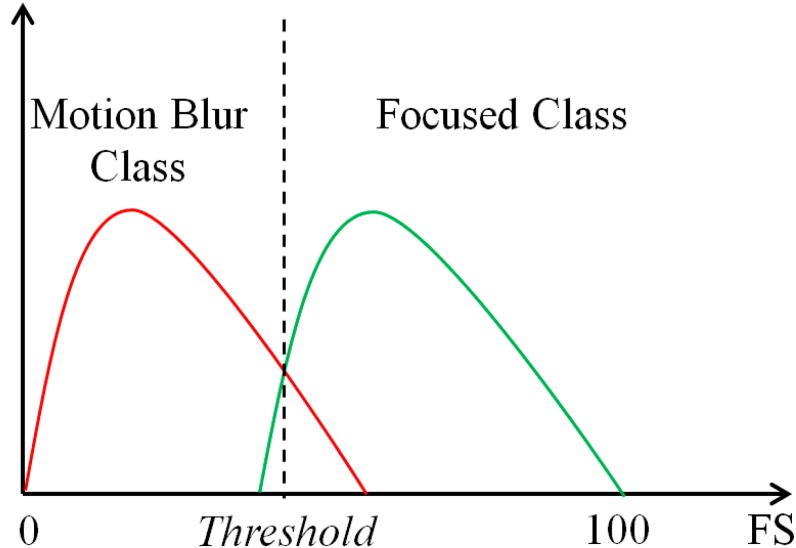
Classification of focused and motion blur classes using proposed focus score measurement (FS).

In Figure 11, we show some examples of focus score measurements of focused and motion-blurred images. Using the proposed focus score measurement method, we can classify the input images into focused or motion-blurred class, as shown in Figure 12. Using the training database, an optimal threshold for classification is determined, with which the minimum classification error is obtained. Then, this optimal threshold is used to classify new input images.

### 2.4. Human Age Estimation Based on MLBP, Gabor Filtering, PCA, and SVR

**Figure 13:**
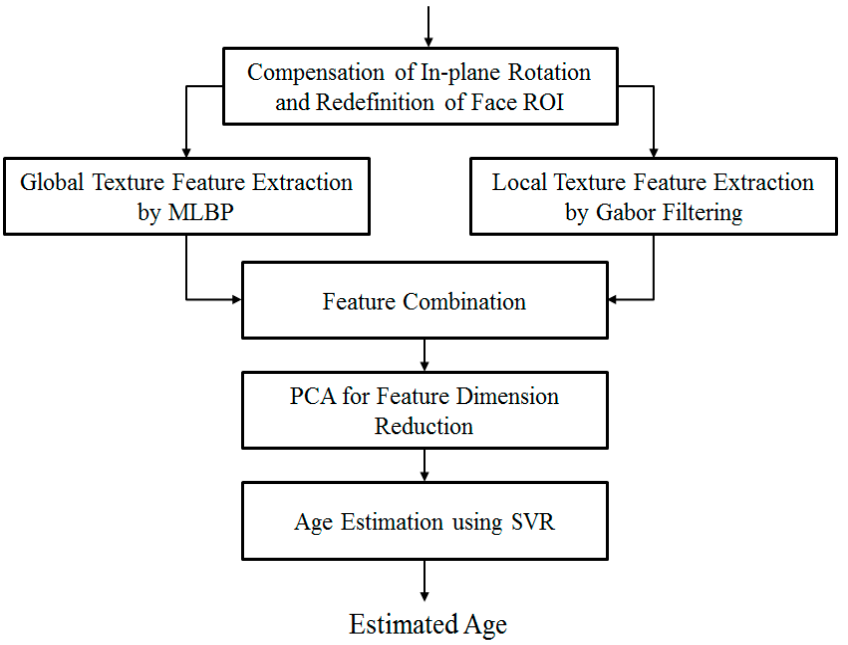
Procedure for age estimation method based on MLBP, Gabor filtering, PCA and SVR.

As described in Section 2.1 and shown in Figure 1, our method uses an age estimation method based on MLBP, Gabor filtering, PCA feature extraction method, and SVR. The detailed procedure of this method is depicted in Figure 13. As explained in Section 2.2, we first perform in-plane rotation compensation using Equation (1). The face ROI detected using Adaboost method normally does not fit the actual face region. Therefore, we perform a further pre-processing step to redefine the face ROI region in order to obtain a more correct face region based on the geometric characteristics of the human face [7]. There are several features that appear on the human face according to human age, such as wrinkles, spots, rough skin, etc. Based on these characteristics, we extract skin features for the estimation problem. There are two types of age feature extraction methods used to extract the age feature, including the global feature extracted by the MLBP method and the local wrinkle feature extracted by the Gabor filtering method [7,14]. In previous research [7], the SVR method is applied directly to the feature combined by MLBP and Gabor filtering. This approach has the limitation of high-dimensional features and noise effects. Therefore, feature dimension reduction and selection of optimal features based on PCA are performed. Finally, we use the SVR method with the PCA features to estimate the human age. Detailed explanations are given in Section 2.4.1 to Section 2.4.3.

#### 2.4.1. Global Age Feature Extraction by MLBP Method

LBP has been used widely in many computer vision systems, such as face description [28], finger-vein recognition [29,30], face recognition [31], facial expression recognition [32], and human age estimation [7,8,9]. This is a powerful method for texture description that offers an image texture descriptor that is robust to illumination and rotation changes. Mathematically, the LBP method is described by Equation (13). In Equation (13), variable *R* indicates the radius of the circle from which the surrounding pixels are taken; variable *P* indicates the number of surrounding pixels; and *s*(*x*) is a thresholding function that takes the value of 1 if the input value is equal to or greater than zero; otherwise, it takes the value of 0. Intuitively, the LBP method encodes each image pixel into a binary code by comparing the surrounding pixels with the center pixel:

(13) $$LBP_{R,P}=\overset{P-1}{\underset{i=0}{\sum }}s\left(g_{i}-g_{c}\right)\times 2^{i}$$

**Figure 14:**
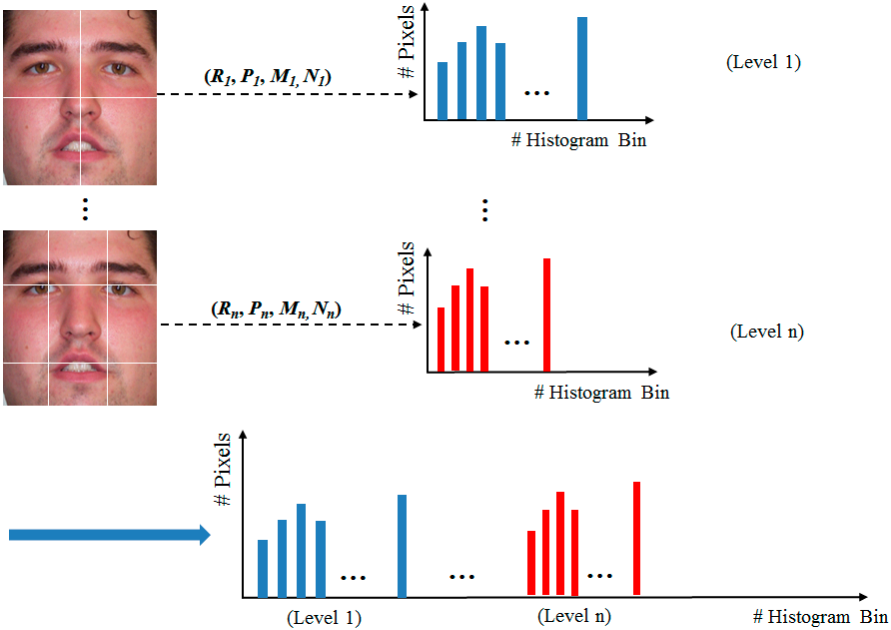
Methodology for feature extraction using MLBP method.

In previous studies [7,8,9,14], the LBP method was used for the human age estimation problem. For this purpose, the LBP codes are first divided into uniform and non-uniform codes. Then, the histogram feature of such codes is acquired and used for age estimation. The uniform codes have the characteristic of at most two bit-wise changes from 0 to 1 (or 1 to 0). This type of LBP code efficiently describes the appearance of micro-texture features of the face, such as wrinkles and spots. The other types of LBP codes that have more than two bit-wise transitions from 0 to 1 (or 1 to 0) are classified as non-uniform codes. These LBP codes represent the very complex texture features normally associated with noise. Therefore, they do not contain sufficient information for age estimation. Consequently, by making a histogram feature of uniform and non-uniform codes, we can represent the characteristic of age features on a human face. The LBP histogram feature of a face ROI is formed by obtaining and concatenating the LBP histograms of many non-overlapped sub-blocks of a face ROI image. In addition, in order to overcome the problem of sub-block size, the MLBP feature is obtained, instead of the LBP feature, as shown in Figure 14 [7].

As shown in Figure 14, the MLBP feature is a histogram feature constructed by concatenating several LBP features, which are obtained by the different parameters of radius (*R*), number of pixels (*P*), and number of sub-blocks (*M_n_*, *N_n_*). Consequently, using the MLBP feature, we can extract the age feature that exploits the richer age information compared with the LBP feature.

#### 2.4.2. Local Age Feature Extraction by Gabor Filtering

Although the MLBP feature works well on making histogram features that describe the appearance of the texture feature, it cannot sufficiently measure the strength of the wrinkle feature. As humans age, the wrinkle feature is presented on some local regions of the face, such as the forehead, left and right sides of the eyes, lower part of the eyes, *etc.* This type of age feature is weak on the face of young people, but it becomes stronger and larger on that of older people. Therefore, our age estimation method uses Gabor filtering to extract the wrinkle feature. Because such feature appears as edges in different directions and sizes according to human age, we use a Gabor wavelet filtering at different scales and directions. Mathematically, Gabor filtering is modeled by the Gaussian function multiplied by a sinusoid wave, as shown in Equation (14) [7,14,33]. In this equation, $\sigma_{x}$ and $\sigma_{y}$ are the standard deviations of the filter in the *x* and *y*-axes, respectively; W is the sinusoid frequency. In our experiment, we use only the real part of Gabor filtering at four scales and six directions to extract the wrinkle feature, as shown in Equation (15):

(14) $$G\left(x,y\right)=\frac{1}{2\pi \sigma_{x}\sigma_{y}}exp\left\{-\frac{1}{2}\left(\frac{x^{2}}{\sigma_{x}^{2}}+\frac{y^{2}}{\sigma_{y}^{2}}\right)+j2\pi Wx\right\}$$

(15) $$R\left(x,y\right)=\frac{1}{2\pi \sigma_{x}\sigma_{y}}exp\left\{-\frac{1}{2}\left(\frac{x^{2}}{\sigma_{x}^{2}}+\frac{y^{2}}{\sigma_{y}^{2}}\right)\right\}\text{cos}\left(2\pi Wx\right)$$

**Figure 15:**
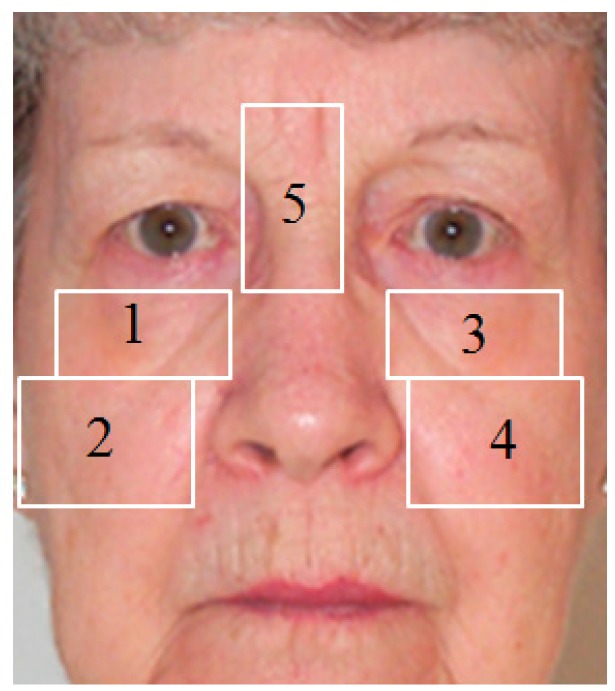
Example of several selected local wrinkle regions to extract wrinkle features.

Based on the detected positions of the two eyes, we first define several local wrinkle regions, as shown in Figure 15. These regions are selected based on where the wrinkle feature normally appears as human age increases. For each selected region, the filtered image is calculated by the convolution operation of the wrinkle region and the Gabor filter. Then, the mean and standard deviation of the filtered image are used as two wrinkle features. In our experiments, we use five local regions and Gabor filtering at four scales and six directions. Consequently, a feature vector in the 240-dimensional space (5 (regions) × 4 (scales) × 6 (directions) × 2 (features)) is obtained to represent the wrinkle feature [7].

#### 2.4.3. Age Estimation by SVR

Using two feature extraction methods, our method obtains two feature vectors, including the MLBP feature vector and Gabor filtering feature vector. The final feature vector is then formed by combining the two feature vectors. In order to perform the feature vector combination, each feature vector is first normalized using the Z-score normalization method. In Equation (16), the values ***µ_i_*** and ***σ_i_*** are the mean and standard deviation vectors of the raw feature vectors ***f_i_*** [7,14], respectively. Then, the combined age feature (***f***) is constructed by concatenating the two normalized feature vectors of MLBP and Gabor filtering using Equation (17):

(16) $${f_{i}}^{*}=\frac{f_{i}-\mu_{i}}{\sigma_{i}}$$

(17) $$f=\left[{f_{1}}^{*}\;,\;{f_{2}}^{*}\right]$$

In previous research [7], the combined feature is used directly as input to the SVR machine to estimate human age. This approach has a limitation of the very high-dimension feature vector and noise effects. As shown in Figure 14, the MLBP feature is constructed by concatenating several LBP feature vectors. Consequently, the MLBP feature is a vector in a very high dimensional space. Processing a high-dimensional feature vector causes the increase of processing time. In addition, the performance of the estimation system can also be affected by redundant information caused by imperfect face ROI estimation and noise. To solve this problem, our research uses the PCA method [34,35,36] to analyze the feature vector, and uses a small number of principal components, instead of all components in the feature vector. Using this scheme, we not only reduce the dimension of the feature vector, but also enhance the performance of the age estimation system by removing some non-important components from the extracted feature vector. The feature vector obtained by PCA is used as input to the SVR machine, and then human age is estimated using SVR. The LibSVM software package was used for implementation in our experiments [37].

## 3. Experiment Results

### 3.1. Description of Database and Performance Measurement

**Figure 16:**
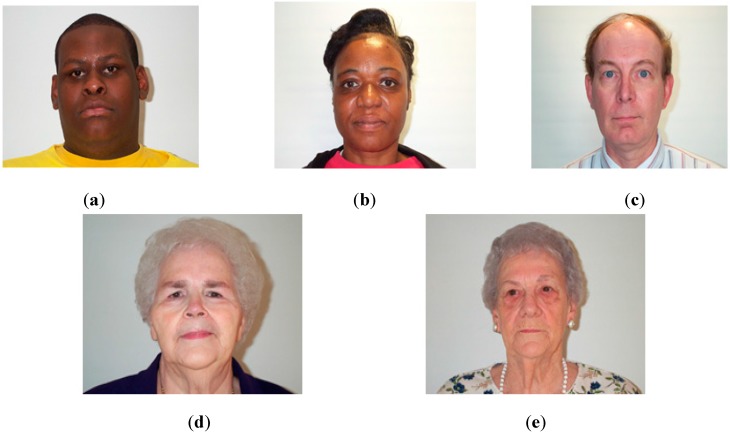
Sample images from PAL database: (**a**) male aged 22; (**b**) female aged 49; (**c**) male aged 52; (**d**) female aged 67 and (**e**) female aged 78.

In this section, we present the experiment results of our methods on motion blur parameters estimation and the age estimation system. For this purpose, we use an open database called PAL [38,39]. The PAL database contains the face images of 580 persons in the age range of 18 to 93 years of different genders (male and female) and races (Caucasian, Africa-American, and others). In order to evaluate the performance of our system, the PAL database is first divided into learning and testing databases twice in order to perform a two-fold cross validation scheme. Described in detail, at each division, half the images are assigned to the learning database, and the other half are assigned to the testing database. Table 2 provides a detailed description of the PAL database and the learning and testing sub-databases. Some sample images from the PAL database are shown in Figure 16.

**Table 2:** Descriptions of PAL database and its learning and testing sub-databases.

Database	Number of Learning Images	Number of Testing Images	Total
Database Part 1	291	289	580
Database Part 2	291	289	580

Because it is extremely difficult to obtain a real motion blur database to test our system, we artificially made the motion blur database using the images from the PAL database. In our research, we assume that the linear motion blur is presented in the face image as shown in Equation (4). Described in detail, for each image in the PAL database, we artificially made the motion-blurred images in four directions (0°, 45°, 90°, and 135°, respectively). In addition, the amount of motion blur (motion length) is varied from 1 (without motion blur) to 15 (with much motion blur) with a step of 2. A detailed description of the motion blur database is given in Table 2. From this motion blur database, we classify the images into three groups: focused, slightly blurred, and blurred. The focused group contains images without motion blur (images from the PAL database without motion blur) and trivial motion blur with motion length of 3. The slightly blurred database contains images with a larger amount of motion blur compared to the focused database with an amount of motion blur of 5, 7, and 9. The other motion-blurred image with amount of motion blur of 11, 13, and 15 are grouped into the blurred database. In total, we obtained a database of 16,820 images (580 (original image) + 580 (images) × 4 (directions) × 7 (motion length values)). This database of 16,820 images is listed in Table 3.

**Table 3:** Description of motion blur database used in our experiments.

Number of Images	Focused Database (Motion Length is 1 and 3)	Slightly Blurred Database (Motion Length is 5, 7, and 9)				Blurred Database (Motion Length is 11, 13, and 15)				Total Number of Images
		0°	45°	90°	135°	0°	45°	90°	135°	
Learning Database	1455	873	873	873	873	873	873	873	873	8439
Testing Database	1445	867	867	867	867	867	867	867	867	8381

The goal of the age estimation system is to accurately estimate human age. This means that the error between the estimated age and ground-truth should be small. In order to measure the performance of the estimation system, our method uses the mean absolute error (MAE) criteria. Mathematically, MAE measures the average estimation error between the estimated and ground-truth ages of the images in the testing database, and it is represented by Equation (18) [6,7,9,14].

In this equation, the value of *N* indicates the number of testing images, and *a_k_* and *a_k_’* are the ground-truth and the corresponding predicted ages, respectively. As indicated in Equation (18), a smaller value of MAE indicates a better estimation performance of the estimation system:

(18) $$MAE=\frac{1}{N}\overset{N}{\underset{k=1}{\sum }}\left|{a_{k}}^{\prime }-a_{k}\right|$$

### 3.2. Performance Evaluation of the Proposed Motion Blur Parameters Estimation

In the first experiment, we evaluate the performance of our method for motion blur parameters estimation and the focus score measurement depicted in Section 2.3. As shown in Equation (12), our method uses the proposed focus score measurement to first separate the focused and motion-blurred images. As shown in Figure 12, using the training database, the optimal threshold for classifying the focused and blurred images is determined. Using this optimal threshold, we classify the input testing images into one of two classes of focused or motion blurred by comparing the focus measurement of the input images with the optimal threshold. Table 4 lists the classification results of our focus score measurement on the two testing databases. In this table, the focused class contains the image without motion blur, and the blurred class contains the images with motion blur effects.

Because of blur effects, nine images failed for face detection in the testing databases. Consequently, a total of 8372 images were used for this experiment, instead of the 8381 images in each testing database, and this includes 289 focused images and 8083 blurred images. For testing database 1, only one image from a total of 289 images of the focused class was misclassified into the blurred class, whereas 14 images from a total of 8083 images from the blurred class were misclassified into the focused class. For testing database 2, these values are three and 14 images, respectively. On average, the classification equal error rate of the two testing databases is 0.433%. Through this experiment results, we can conclude that our focus score measurement method for motion blur assessment is efficient for the classification of focused and motion-blurred images.

**Table 4:** Classification results of images into focused and blurred classes for two testing databases.

Number of Images (Testing Database 1/Testing Database 2)	Focused Class (Images without Motion Blur)	Blurred Class (Images with Motion Blur)
Focused class (Images without motion blur)	288 (99.654%)/286 (98.962%)	1 (0.346%)/3 (1.038%)
Blurred Class (Images with motion blur)	14 (0.173%)/14 (0.173%)	8069 (99.827%)/8069 (99.827%)

In the next experiment, we measure the performance of our estimation method for motion blur parameters. As indicated in Table 3, we artificially made the blur image database using four major directions: 0°, 45°, 90°, and 135°, respectively. In addition, the amount of motion blur is varied from 3 to 15, which corresponds from trivial to significant blur. In total, 16,240 images (580 (original images) × 7 (motion lengths) × 4 (directions)) were used in this experiment. Table 5 lists the average estimation error of the motion direction and amount of motion blur. As indicated in this table, the average error of the direction estimation is approximately 0.709° and the average error of the estimation of the amount of motion blur is approximately 0.309. From this, we can confirm that our method can correctly estimate the direction and amount of motion blur.

**Table 5:** Estimation errors of motion blur parameters using entire motion blur database.

Average Error of Direction Estimation	Average Error of Amount of Motion Blur (Motion Length)
0.709°	0.309

**Figure 17:**
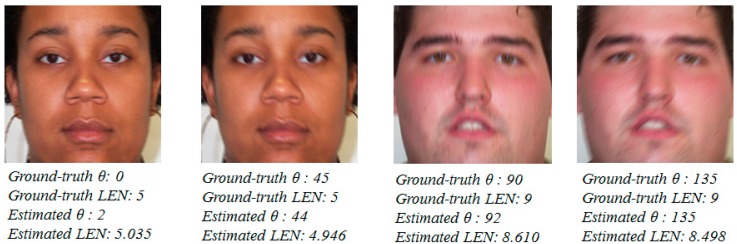
Examples of estimation results of motion blur parameters.

In Figure 17, we show some examples of the estimation results of the motion direction and amount of motion blur. By comparing the ground-truth parameters and estimation results, we can see that the proposed estimation method for motion blur parameters works well in all the cases of images. In this figure, θ means the motion direction, and *LEN* means the amount of motion blur on the given images.

### 3.3 Performance Evaluation of Proposed Age Estimation Method

In order to obtain the best estimation performance, age estimation systems are normally trained using good quality images. For the testing phase, the input face image is also required to be of good quality. This requirement is necessary because poor quality images do not contain sufficient information for the estimation task. Therefore, if a poor quality image is used with an age estimation system, the consequent estimation result becomes untrustworthy. In order to demonstrate the effects of motion-blurred images on the age estimation system, we first perform an experiment on the age estimation system with motion-blurred images by measuring MAE of the age estimation system using the motion blur database from Table 3 and the age estimator that uses the focused images. In this experiment, the database from Table 3 is separated manually for the purpose of demonstrating the effects of motion blur on the age estimation system. Detailed estimation results are listed in Table 6 with each sub-database according to motion direction and amount of motion blur.

**Table 6:** Estimation results of motion blur database without our proposed age estimation method.

MAE	Original PAL Database	Focused Database (*LEN* = 1, 3)	Slightly Blurred Database (*LEN* = 5, 7, 9)				Blurred Database (*LEN* = 11, 13, 15)				Average MAE
			0°	45°	90°	135°	0°	45°	90°	135°	
Testing Database 1	5.89	6.45	8.90	9.89	8.22	10.67	12.58	13.14	10.90	14.11	10.26
Testing Database 2	6.15	6.40	8.17	9.07	7.36	9.79	11.25	11.87	9.90	12.95	9.42
Average MAE of Entire Database	6.02	9.87									

As indicated in this table, the average error (MAE) of the estimation system without motion-blurred images is 6.02 years. With motion-blurred images, the error increases according to the direction and amount of motion blur. On average, a MAE of 9.87 years is obtained from the entire motion blur database, which is much higher than the MAE of 6.02 years for the system using only the focused (good quality) images. From this result, we can conclude that the motion-blurred images have very strong effects on the age estimation system and result in degradation of the estimation performance.

To solve the problem of motion blur effects on age estimation systems, as depicted in Figure 1, our method pre-classifies the motion-blurred images into one of several groups of motion blur direction and amount of motion blur. Based on this result, human age is estimated using an appropriate age estimator for each group.

In the next experiment, we use the proposed estimation method of motion blur parameters to estimate the motion direction and amount of motion blur in input images. We classify the input images into one of several groups of motion blur based on the estimation results of the motion blur parameters. Using the motion blur database from Table 3 and our method, the detailed experiment results of the pre-classification step are indicated in Table 7. In this experiment, we pre-classified the images into nine groups, including the focused groups that contain the focused images and trivial motion blur (the amount of motion is one and three, where one indicates no motion blur), and the other eight blur groups according to motion direction and amount of motion blur. As indicated in the table, with the exception of some images that failed for face detection, all the images are classified correctly into the corresponding groups. This experiment result proves that our estimation method for motion blur parameter is efficient for correctly estimating the motion direction and amount of motion blur.

**Table 7:** Estimation results of motion blur database using our estimation method for motion blur parameter.

Classification Rate (%) (Testing Database 1/ Testing Database 2)		Focused Database (*LEN* = 1, 3)	Slightly Blurred Database (*LEN* = 5, 7, 9)				Blurred Database (*LEN* = 11, 13, 15)			
			0°	45°	90°	135°	0°	45°	90°	135°
Focused Database (*LEN* = 1, 3)		100/100	0/0	0/0	0/0	0/0	0/0	0/0	0/0	0/0
Slightly Blurred Database (*LEN* = 5, 7, 9)	0°	0/0	100/100	0/0	0/0	0/0	0/0	0/0	0/0	0/0
	45°	0/0	0/0	100/100	0/0	0/0	0/0	0/0	0/0	0/0
	90°	0/0	0/0	0/0	100/100	0/0	0/0	0/0	0/0	0/0
	135°	0/0	0/0	0/0	0/0	100/100	0/0	0/0	0/0	0/0
Blurred Database (*LEN* = 11, 13, 15)	0°	0/0	0/0	0/0	0/0	0/0	100/100	0/0	0/0	0/0
	45°	0/0	0/0	0/0	0/0	0/0	0/0	100/100	0/0	0/0
	90°	0/0	0/0	0/0	0/0	0/0	0/0	0/0	100/100	0/0
	135°	0/0	0/0	0/0	0/0	0/0	0/0	0/0	0/0	100/100

Based on these pre-classification results, we performed the age estimation method for images in each group by applying an appropriate age estimator for each group. As explained in Section 2.4, after pre-classifying the motion-blurred images into appropriate groups, human age is estimated using a suitable age estimator for that group. For this purpose, the training process is first done using the images that correctly belong to each group of motion-blurred images. Then, the trained age estimators are used to estimate human age in the input test images. Detailed estimation results using our method are indicated in Table 8. On average, we obtained a MAE value of 6.48 years. Although this MAE value is slightly higher than the MAE of 6.02 years for the system using only the focused good quality images (Table 6), this MAE value is much lower than the MAE of 9.87 years for the system without our method (Table 6).

This result is caused by the poor quality of images caused by the motion blurring effects. Because of motion blurring, the quality of the face image is reduced and some spurious age feature is presented on the face region. Consequently, age information in the face region is lost or not represented correctly. Therefore, although we trained an age estimator suitable for each group of motion-blurred images, the estimation performance of the blur groups cannot be as good as the estimation performance of the focused good quality group.

For this reason, the performance of our method cannot improve compared with the system without motion blur effects. However, the performance of our method is superior to that of the system that does not consider the effects of motion blurring.

**Figure 18:**
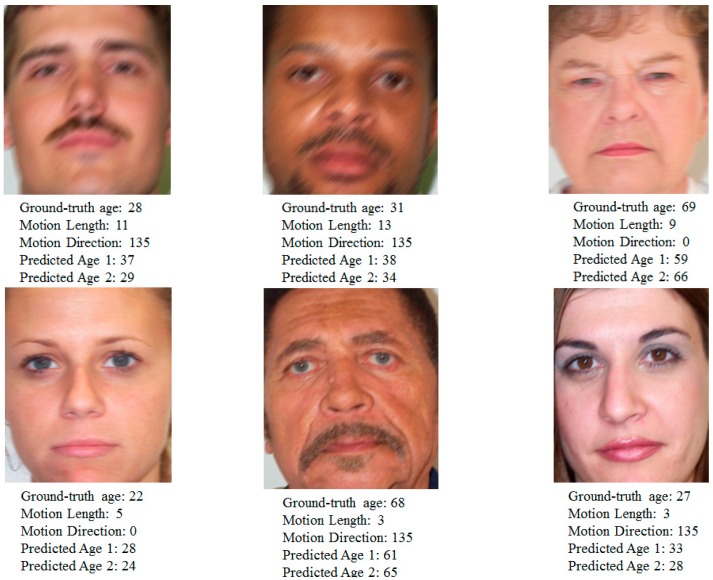
Sample results of estimation process with and without our method.

In Figure 18, we show some sample results of the age estimation system with and without our method. In this figure, “Predicted Age 1” means the age estimation result of the system that does not consider the effects of motion blurring; “Predicted Age 2” means the estimation result of our method that considers the effects of motion blurring. It can be seen from these examples that our age estimation method produces better estimation results compared with the system without our method.

**Figure 19:**
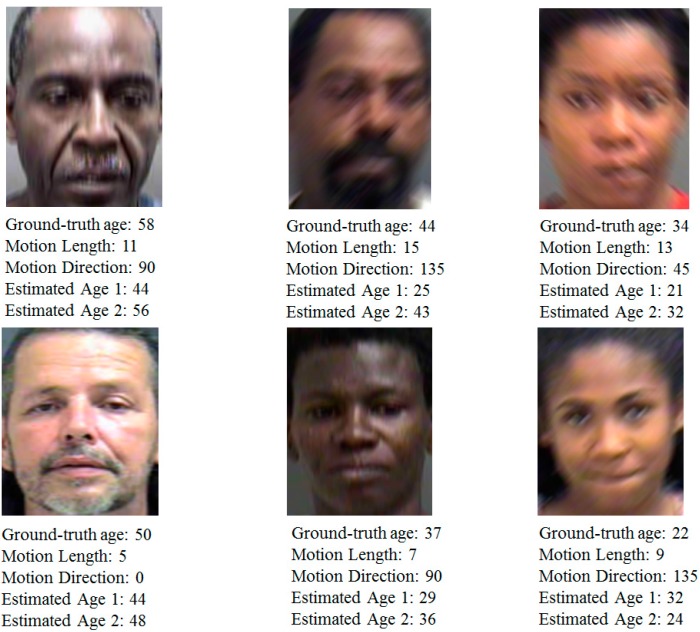
Sample results of estimation process with and without our proposed method using the new motion blur database obtained from MORPH database. (Estimated Age 1 means the age estimation result of the system that does not consider the effects of motion blurring; Estimated Age 2 means the estimation result of our method that considers the effects of motion blurring).

We performed the additional experiments with the second evaluation dataset of MORPH. The MORPH is composed of over 55,000 images from over 13,000 people from 16 years old to 77 years old [40]. From this database, we randomly select images at different age, gender and individuals for our new experiments. Consequently, a new motion blur database of 17,400 images that is composed of 600 focused images and 16,800 motion-blurred images (600 (focused images) ×4 (directions) × 7 (motion lengths)) is constructed for our new experiments. The experimental results are included in Table 9, Table 10, Table 11, Table 12 and Table 13 and Figure 19. As shown in Table 3, Table 4, Table 5, Table 6, Table 7 and Table 8 by PAL database and Table 9, Table 10, Table 11, Table 12 and Table 13 by MORPH dataset, the accuracies of age estimation by our method are similar in these two databases, which can confirm the generalization of our method in different databases.

**Table 8:** Estimation performances of our age estimation method using motion blur databases.

MAE	Focused Database (*LEN* = 1, 3)	Slightly Blurred Database (*LEN* = 5, 7, 9)				Blurred Database (*LEN* = 11, 13, 15)				Average MAE
		0°	45°	90°	135°	0°	45°	90°	135°	
Testing Database 1	5.88	6.10	6.63	6.16	6.60	6.29	6.75	6.18	6.61	6.54
Testing Database 2	6.14	6.18	6.47	6.25	6.68	6.36	6.62	6.31	6.87	6.41
Average of Entire Database	6.48									

**Table 9:** Description of the new motion blur database obtained from MORPH database used in our experiments.

Number of Images	Focused Database (Motion Length is 1 and 3)	Slightly Blurred Database (Motion Length is 5, 7, and 9)				Blurred Database (Motion Length is 11, 13, and 15)				Total Number of Images
		0°	45°	90°	135°	0°	45°	90°	135°	
Learning Database 1	1515	909	909	909	909	909	909	909	909	8787
Testing Database 1	1485	891	891	891	891	891	891	891	891	8613
Learning Database 2	1505	903	903	903	903	903	903	903	903	8729
Testing Database 2	1495	897	897	897	897	897	897	897	897	8671

**Table 10:** Classification results of images into focused and blurred classes for two testing databases using new motion blur database obtained from MORPH database.

Number of Images (Testing Database 1/Testing Database 2)	Focused Class (Images without motion blur)	Blurred Class (Images with motion blur)
Focused class (Images without motion blur)	297 (100.000%)/299 (100.000%)	0 (0.0%)/0 (0.0%)
Blurred Class (Images with motion blur)	13 (0.157%)/13 (0.156%)	8273 (99.843%)/8323 (99.844%)

**Table 11:** Estimation errors of motion blur parameters using entire new motion blur database obtained from MORPH database.

Average Error of Direction Estimation	Average Error of Amount of Motion Blur (Motion Length)
0.837°	0.332

**Table 12:** Estimation results of motion blur database without our proposed age estimation method.

MAE	Original MORPH Database	Focused Database (*LEN* = 1, 3)	Slightly Blurred Database (*LEN* = 5, 7, 9)				Blurred Database (*LEN* = 11, 13, 15)				Average MAE
			0°	45°	90°	135°	0°	45°	90°	135°	
Testing Database 1	5.99	6.36	8.18	9.65	7.82	10.12	10.18	11.29	9.78	11.81	9.25
Testing Database 2	6.02	6.42	9.10	12.29	9.51	12.72	13.18	16.08	13.29	16.84	11.76
Average MAE of Entire Database	6.01	10.51									

**Table 13:** Estimation performances of our age estimation method using motion blur databases.

MAE	Focused Database (*LEN* = 1, 3)	Slightly Blurred Database (*LEN* = 5, 7, 9)				Blurred Database (*LEN* = 11, 13, 15)				Average MAE
		0°	45°	90°	135°	0°	45°	90°	135°	
Testing Database 1	5.90	6.00	5.85	5.86	6.12	6.23	6.33	6.34	6.17	6.08
Testing Database 2	5.76	5.76	6.07	5.75	5.98	6.30	6.38	6.06	6.16	6.01
Average of Entire Database	6.05									

**Figure 20:**
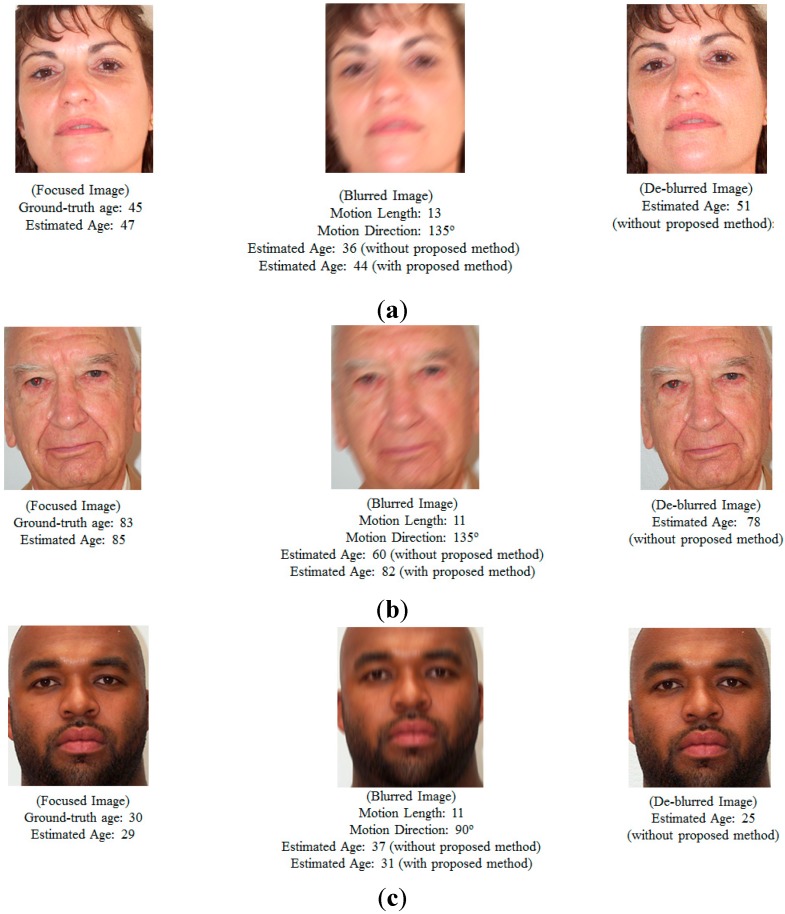
Examples of age estimation results in case of focused images, motion blur images with and without our proposed method and the de-blurred images using Wiener filter.

As the next experiment, we compared the performance by age estimation after de-blurring filter (Wiener filter) with that by our proposed method on PAL database. As shown in Table 8 and Table 14, we can confirm that the accuracy of age estimation by our method is higher than that by age estimation after de-blurring filter. The reason why the accuracy of age estimation after de-blurring filter is lower than that by our method is that the additional noises can be included by the de-blurring filter or the blurred image is not completely restored to the focused one by the de-blurring filter as shown in Figure 20.

**Table 14:** Age estimation accuracies using de-blurring filter (Wiener filter) on motion-blurred images of testing database 1 and 2.

Testing Database 1	Testing Database 1	Average MAE
7.818	8.081	7.950

In our experiment, we just consider the motion blurring of linear type, and use the assumption that the motion blurring of our experimental images does not include the two (or more than two) directions or degree of motion blurring. As shown in Table 3, Table 4, Table 5, Table 6, Table 7 and Table 8 and Table 9, Table 10, Table 11, Table 12 and Table 13, we show that our method can accurately estimate the age with the motion blurred images of two databases where various degree of blurring (from 1 to 15) and various directions of blurring (vertical (0°), horizontal (90°), diagonal (45°), anti-diagonal (135°)) are included. From that, we can confirm that our method can generalize in various cases of motion blurring.

## 4. Conclusions

In this paper, we proposed a new human age estimation method that is robust to the effects of motion blurring. In general, motion blurring can occur on captured images because of camera movement and/or the movement of the viewed objects. Because of this effect, the age feature of the face can be changed according to the amount of motion blur and direction, which can cause performance degradation in age estimation systems. In order to make the age estimation system robust to the effects of motion blurring, the parameters of motion blurring (amount of motion blur and motion direction) were first estimated using our proposed estimation method. By estimating the motion parameters, we pre-classified the images into one of several groups of motion blurring according to the estimated amount of motion blur and motion direction. Finally, an appropriate age estimator for each group of motion blurring based on MLBP, Gabor filtering, PCA, and SVR was used to estimate human age. For future work, we plan to investigate other effects of low image resolution, low light, or image distortion on age estimation systems in order to enhance the performance of age estimation systems. In addition, we would perform the experiments with other real database of motion blurring.
